# Linking quantitative genetics with community-level performance: Are there operational models for plant breeding?

**DOI:** 10.3389/fpls.2022.733996

**Published:** 2022-10-19

**Authors:** Cyril Firmat, Isabelle Litrico

**Affiliations:** ^1^AGIR, INRAE, University of Toulouse, Castanet-Tolosan, France; ^2^P3F UR 004, INRAE, Le Chêne RD150, Lusignan, France

**Keywords:** community genetics, eco-evolutionary dynamics, breeder’s equation, agroecology, anticipatory predictions, general mixing ability (GMA), specific mixing ability (SMA), genetic variance

## Abstract

Plant breeding is focused on the genotype and population levels while targeting effects at higher levels of biodiversity, from crop covers to agroecosystems. Making predictions across nested levels of biodiversity is therefore a major challenge for the development of intercropping practices. New prediction tools and concepts are required to design breeding strategies with desirable outcomes at the crop community level. We reviewed theoretical advances in the field of evolutionary ecology to identify potentially operational ways of predicting the effects of artificial selection on community-level performances. We identified three main types of approaches differing in the way they model interspecific indirect genetic effects (IIGEs) at the community level: (1) The community heritability approach estimates the variance for IIGE induced by a focal species at the community level; (2) the joint phenotype approach quantifies genetic constraints between direct genetic effects and IIGE for a set of interacting species; (3) the community-trait genetic gradient approach decomposes the IIGE for a focal species across a multivariate set of its functional traits. We discuss the potential operational capacities of these approaches and stress that each is a special case of a general multitrait and multispecies selection index. Choosing one therefore involves assumptions and goals regarding the breeding target and strategy. Obtaining reliable quantitative, community-level predictions at the genetic level is constrained by the size and complexity of the experimental designs usually required. Breeding strategies should instead be compared using theoretically informed qualitative predictions. The need to estimate genetic covariances between traits measured both within and among species (for IIGE) is another obstacle, as the two are not determined by the exact same biological processes. We suggest future research directions and strategies to overcome these limits. Our synthesis offers an integrative theoretical framework for breeders interested in the genetic improvement of crop communities but also for scientists interested in the genetic bases of plant community functioning.

## Introduction

Ongoing agricultural intensification, which started during the last century, has successfully increased yields by relying on large-scale monoculture. But it is now generally accepted that this form of agriculture is not sustainable and does not ensure stable food supplies in the current context of high environmental variability (Lin et al., 2008). Field experiments on grassland communities in the two last decades have established that species diversity is a strong determinant of yield, yield stability, and other agroecosystem functions (Hector et al., 1999; Tilman et al., 2001; Isbell et al., 2015). Congruent benefits have been obtained for cereal–grain legume intercrops (Hauggaard-Nielsen et al., 2008; Bedoussac et al., 2015; Annicchiarico et al., 2019; Demie et al., 2022). Together, these results suggest that, compared to monoculture, multispecies cover crops could be a way to maintain and stabilize high yields while reducing the demand for chemical inputs. Overall, growing several crop species in the same plot, either through intercropping, the use of companion crops, or agroforestry, is now among the most promoted strategies to develop sustainable and efficient farming systems (Beillouin et al., 2019).

Today, most efforts invested in plant breeding focus on improving a single species, ignoring the effect of ecological interactions with any other species potentially sown together. This dominant breeding strategy only considers ecological interactions of the improved cultivars in the field among conspecifics. This is questionable for two main reasons. First, well-established positive relationships between plant species richness and agroecosystem functioning (synthesis in Isbell et al., 2017) point to the fact that adding the right species to a monoculture can increase yield and other agronomically relevant parameters. Second, going on improving crops in pure stands if the target environment is a diversified cropping system is expected to substantially reduce the efficiency of breeders’ actions (Annicchiarico et al., 2019). In these cases, continuing breeding in the same way as today might cause a lock-in in the transition toward more agroecological practices, making the situation problematic for the plant breeding sector.

Litrico and Violle (2015) proposed that redesigning breeding methods accounting for species interactions and their underlying traits could enable an upward shift of the typical saturating curve that links ecological functions and species richness in natural communities (refer to Tilman et al., 2014). Thus, the plant breeder’s usual problem, i.e., “*What is the best way to improve the performance of this species from this gene pool?”* should become “*What is the best way to improve the performance of this plant community* (refer to Box 1) *by selecting within several gene pools?”* (refer to Box 2 for some illustrative examples). Regarding biological knowledge, this is far from a minor transition, as the second question requires coupling two biodiversity scales, the population genetic scale and the ecological community scale. The properties of a complex and composite structure cannot be trivially extrapolated from the properties of its components (Anderson, 1972). Typically, the properties of a plant community cannot be trivially extrapolated from the genetic composition of its individual component species. Such an extrapolation remains a key challenge in applied evolutionary and ecological research (Levin, 1992).

**BOX 1 Definitions of specific terms used in the text.**

**Plant community:** Synonym for intercropping systems or mixtures of species used in the context of this review. Assemblage of plant species (two or more) growing in the same place and interacting through processes such as competition, facilitation or resource partitioning. These interactions and their variability generate functional properties measurable at the scale of the whole community (refer to examples in Box 2).

**Community-level variable (*c*)**: The target of selection predefined by the breeder according to agronomic objectives. Its variation is expected to result from both *direct (DGE)* and *interspecific indirect (IIGE) genetic effects* that occur within the *plant community*. It is modeled as a linear selection index. For two species (*A* and *B*) in the mixture, each with *n* traits: *c* = (*z*_*A1*_ + *z*_*A2*_ + … + *z*_*An*_) + (*z*_*B1*_ + *z*_*B2*_ + … + *z*_*Bn*_). Specific cases of *c* can simplified to the yield measurements of a single *associated species* of economic interest in the mixture (thus, e.g., *c* = *z*_*A1*_). The phenotype *z*_*A1*_ of this species is expected to be affected by *IIGE* from the genotypes of one or several species in the mixture.

**Indirect genetic effect (IIGE):** The expression of genes in one species affecting the phenotypic expression in individuals of another species and potentially the value of the functional trait (i.e., the *community-level variable c*) at the whole community level. It is therefore distinct from the usual direct genetic effects (DGE) qualifying the expression of genes on the phenotype of the individual bearing these genes.

**Focal species:** A species for which genetic relatedness is experimentally known to measure the variation in its indirect genetic effect (i.e., the IIGE) on a *community-level variable* (*c*) or on the phenotype of an *associated species*. This makes it possible to select genotypes of this focal species based on their IIGE of interest.

**Associated species:** A species grown in interaction with a *focal species*. Phenotypes of this species are measured but genetic relatedness among its individuals may remain unknown in the experimental design.

**BOX 2 Some examples of agronomic goals that could be achieved using breeding approaches that account for interspecific indirect genetic effects.**

To connect the model variables used in the text with agronomic problems, below we use letters in parentheses to identify: the community-level variable as a final goal for the breeder [*c*] and the candidate traits [*z*] assumed to affect it, as measured in a species of the sown community (see main text for details).

**Persistence of legumes in sown grasslands.** In low input multispecies grasslands, the persistence of legume species [*z*] in the cover is a major determinant of the effects of biodiversity on yield (Brophy et al., 2017), and legumes are generally the most sensitive component. Persistence is a heritable trait that can be improved by selection (Smith and Kretschmer, 1989; Casler and Van Santen, 2010; Annicchiarico et al., 2019). Although community performance such as multiyear biomass production and quality [*c*] is the final objective of selection, we have no direct indication of the effectiveness of breeding programs for legume persistence at the grassland community level. Accounting for IIGE linked to the legume persistence trait at the community level and above (e.g., N release that enhances the overall performance of the cropping system) could improve the agronomic relevance of such breeding efforts.

**Weed suppression in faba bean.** Faba bean is among the most promising species in temperate areas to develop to increase protein autonomy, but is quite sensitive to competition with weeds. Intercropping faba bean with cereals is a sound management strategy to solve this problem (Jensen et al., 2010). In barley, resistance to weed competition is known to vary among cultivars (Dhima et al., 2000). Consequently, breeding cereals for increased weed suppressing effect [*z*] can be an IIGE to target to improve faba bean grain yield in no herbicide systems [*c*].

**Lodging reduction in sensitive grain legumes.** Lentil or pea is intercropped by farmers with cereals or with Brassicaceae to avoid legume lodging and to improve their mechanical harvestability (Cowell et al., 1989; Viguier et al., 2018). The rigidity of the stem of the non-legume companion species [*z*] thus becomes a trait of interest for legume performance and harvestability [*c*]. Stem rigidity is known to be genetically variable and, accordingly, performance in intercropping can differ among cultivars (e.g., Cowell et al., 1989). Selecting for this trait can produce a positive response at the scale of the intercrop, through an IIGE on the legume component. In addition, the lodging resistance traits of the legume lose their relevance under intercropping (Hauggaard-Nielsen and Jensen, 2001), allowing breeders to invest efforts in other traits more closely related to yield performance.

As a professional practice rooted in quantitative genetics, the original objective of plant breeding was and continues to be population-level variation. The quantitative genetic bases of species interactions and community properties were thus broadly excluded from the rich history of modeling efforts (refer to e.g., Walsh and Lynch, 2018). Although the implications of ecological interactions among individuals within species have been well studied by quantitative geneticists (Griffing, 1967; Moore et al., 1997; Wolf et al., 1999; review in Bijma, 2014), this does not address the coupling between two nested diversity scales as interspecific indirect genetic effects (hereafter IIGE, refer to Box 1). The evolutionary dynamics of IIGE do not involve the same processes as indirect genetic effect *within* species as the latter is assumed to account for individual relatedness (Queller, 2014). The study of IIGE needs to assume that individual relatedness is null and its effects can be treated as an external environmental influence whose variability is partly determined by genes (Goodnight, 1991).

**FIGURE 1:**
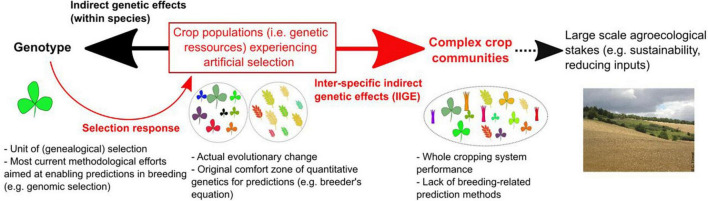
The context of the present survey and its scope (represented by red arrows and texts in the figure). Current plant breeding activities can be viewed as lying under a double-constraint between, on the one hand, technological advances that favors predictions at the genotypic level (black arrow pointing left) and, on the other hand, broad-scale agroecological stakes requiring predictions of plant breeding effects at much higher biological levels (red arrows pointing right). Population level, as the level of evolutionary changes (i.e., the whole set of genetic resources that can be controlled by breeders), lies at the core of this double-constraint. The bold red arrow indicates the focus of the present survey, i.e., models predicting changes in the performances of a complex crop community resulting from any type of breeding-induced evolutionary changes.

The scope of the present article (Figure 1) is to explore recent modeling efforts conducted under the umbrella of eco-evolutionary dynamics (Hendry, 2016) from a plant breeding perspective. This exploration can be an inspiring source of concepts and models to extend the practice of plant breeding to the crop community scale, i.e., the minimum scale of interest from an agroecological point of view. While evolutionary biology (as well plant breeding) was for many years focused on the effect of natural (or artificial) selective factors on evolution (or “genetic improvement”), eco-evolutionary approaches have also investigated the effects of evolutionary changes in the environment. Eco-evolutionary thinking addresses the problem of the relationship between populations and their environment as a feedback loop between ecology and evolution (Schoener, 2011). It is therefore potentially relevant for the challenge facing community-level plant breeding today. The first task is to provide breeders with genetic models to help them to identify the range of community-level breeding problems. In this review, we postulate that the “evo-to-eco” half of the eco-evolutionary causal circle can provide some of the missing elements required to renew plant breeding practices and orient them toward more integrative and sustainable objectives.

As stressed by Casler and Van Santen (2010), plant breeders often have to make difficult choices based on incomplete scientific knowledge. Current plant breeding practice mostly pursues continuous improvement of predefined value criteria of a given cultivar (i.e., through the notions of ideotype and breeding indices). Accounting for genetic diversity and species interactions to improve mixed cropping systems requires accounting for the distributions of traits to identify the best performing genotypes (Litrico and Violle, 2015). The breeder’s choices become even more difficult and knowledge even more incomplete as the biological system to be managed becomes substantially more complex. We consider that selection theory is a powerful tool for objectivizing daily breeding practice and appraising its future orientations (Cobb et al., 2019). In the present context, we need to further extend this theory to shed light on the community-level consequences of choosing certain genotypes within a range of genetic resources. We thus aim to describe models that provide guidance for choosing the best strategy when breeding targets include interacting species. To guarantee the practical relevance of our survey, we focused on models based on the breeder’s equation or its parameters (Walsh and Lynch, 2018; see details at the end of this section). Modeling approaches based on more complex models that are usually designed for the purpose of disentangling eco-evolutionary causal feed-back in the wild (typically: Ellner et al., 2011) are consequently not relevant for our purpose here.

We first distinguished “evo-to-eco” approaches in the literature according to the concepts they use to interface the two biodiversity scales: the population-level response to selection and a community-level variable. Thus, for each approach, we start by providing the assumed underlying statistical equation for interfacing the community variable with quantitative genetic effects. We then discuss whether each approach is potentially “practically operational” when used by practitioners to manage crop genetic resources (i.e., breeders, but also certain farmers and crop conservationists). Here, we define “practically operational” as fulfilling three criteria: first, model parameters are expected to be estimated or approximated from data routinely obtainable by practitioners or with reasonable additional experimental efforts and no loss of effectiveness in their breeding activities; second, these models should be meaningful in the sense that they rely on biologically realistic assumptions and make predictions based on a theoretically sound framework (sensu Houle et al., 2011), here, the breeder’s equation; third, we expect the modeling approaches to provide practitioners with *anticipatory predictions*.

### Description of the modeling approaches

#### Epistemological remark

Below we follow Maris et al. (2018)’s enlightening distinction between anticipatory and corroboratory predictions. Corroboratory predictions are hypothesis-derived predictions. Their goal is to be compared to observations for the *purpose of understanding*. They are necessary for corroborating or invalidating a hypothesis. In this paper, we analyze how quantitative genetic models of selection across two levels of diversity can provide operational anticipatory predictions. Anticipatory predictions consist in the application of extant knowledge. They can be used to achieve a *transformative goal* (Maris et al., 2018), i.e., the essence of plant breeding. Indeed, breeders usually have to act on a genetic system before knowing exactly how the system they are acting on will react (i.e., before knowing its response to selection). In this context, anticipatory predictions are expected to fuel a decision-making process by improving the intelligibility of the potential effects of breeders’ practices on complex, multispecies genetic systems. For this purpose, the three criteria mentioned in the previous section are tightly interlinked.

#### Notation

The breeder’s equation and its equivalent forms (Walsh and Lynch, 2018) predict the mean population change in a trait *z* from the product of a measure of genetic variation (such as heritability) by a measure of selection strength (such as a selection differential, *S*):


(1) Δ⁢z¯=h⁢S2

with the heritability h=2σG2σP2, i.e., the ratio of the (additive) genetic to total phenotypic variance of the measured trait. This simple formula is widely used by breeders and evolutionary biologists but under several equivalent forms (cf. Walsh and Lynch, 2018; Bijma, 2020). Breeders emphasize the selection intensity $\bar{i}$ and the accuracy of selection *h*:


(2) $$\Delta \,\bar{z}=\sigma_{G}\,\left(\frac{\sigma_{G}}{\sigma_{P}}\right)\,\left(\frac{S}{\sigma_{P}}\right)=\sigma_{G}\,h\,\bar{i}$$

while the equivalent version by Lande (1979) is most commonly used in evolutionary biology:


(3) $$\Delta \,\bar{z}=\sigma_{G}^{2}\,\left(\frac{S}{\sigma_{P}^{2}}\right)=\sigma_{G}^{2}\,\beta$$

with β the linear selection gradient, i.e., the slope of the linear regression of the relative fitness *w* on trait *z* (β = cov[*z*, *w*]/var[*z*]). This last version (eq. 3) can be extended to predict the change in the multivariate case through the concepts of G-matrix and multivariate selection gradient **β** (Lande, 1979):


(4a) $$\Delta \,\bar{\text{z}}=\text{G}\,\beta$$

or, under a developed matrix form for *l* traits:


(4b) $$\Delta \,\bar{\text{z}}=\left[\begin{array}{cccc} G_{11} & G_{12} & \ldots & G_{1\,l}\\ G_{21} & G_{22} & \ldots & G_{2\,l}\\ \vdots & \vdots & & \vdots \\ G_{l\,1} & G_{l\,2} & \ldots & G_{l\,l} \end{array}\right]\,\left[\begin{array}{c} \beta_{1}\\ \beta_{2}\\ \vdots \\ \beta_{\text{l}} \end{array}\right]$$

with G_*ii*_ the genetic variance for the *i*^th^ trait and *G*_*ij*_ its genetic covariance with the *j*^th^ trait, and *β_i_* the selection gradient for the *i^th^* trait. By focusing on the case of artificial selection for plant breeding, we assume that selection is strong, and that β is controlled by the breeder, therefore known with high accuracy. Consequently below, the error due to the incidental influence of natural selection on potentially unmeasured traits (synthesis in Walsh and Lynch, 2018, Chapter 20) is treated as negligible.

In the following, depending on the approach described, we refer to each or all of the three equivalent formulations mentioned above (eqs. 1, 2, 3, or 4) to extend the effects of genetic evolution (in response to artificial selection) on a community-level variable. Below, this *community-level variable* is labeled *c* (refer to Box 1 for a definition and refer to Box 2 for biological examples). *c* potentially encompasses several components of community functions such as the total biomass yield of mixtures of perennial forage crops, or the yield of cereal grain when a cash crop and a companion forage legume are selected together, or any other ecological functions such as weed suppressing effects among grain legumes when a cereal species is intercropped for this purpose (refer to the example of faba bean in Box 2). As we will see below, *c* can be a community-level index including weighted performance components from several species and functions.

Our focus is on the links between the population and the community levels. Thus, we do not distinguish between the type of breeding scheme or methods, which are well summarized elsewhere (cf. Walsh and Lynch, 2018). We thus made our analytical framework as general as possible, so it can be used for different artificial (i.e., intentional) multispecies selection approaches based on randomized trials with available estimates of genotypic or phenotypic values for selection candidates to produce the next generation. It is therefore able to cover a wide a range of breeding strategies and tools depending on how the quantitative genetic parameters are estimated (e.g., mass, family-based, genomic selection). Our analytical framework can therefore be easily adapted to account for breeding cycle length, plant reproductive systems (self- and cross-pollinated crops), or any other parameters that affect breeding efficiency, for instance in simulation approaches (Bančič et al., 2021).

### Approach 1: Estimating the heritability of interspecific indirect genetic effects

#### Generalities and theoretical grounding

The most straightforward and intuitive approach to examine how genetic variation affects a variable *c* at the community level is to apply the standard quantitative genetic expression to the variable concerned:


(5) $$c_{i\,k}=\mu +g_{B\,i}+e_{i\,k}$$

This assumes that the value of *c* for the *i^th^* community comprised of associated species (refer to definition in Box 1) will deviate from its mean *μ*, under the influence of the genotype *g*_*Bi*_ of a focal species *B* (Box 1) and under the influence of environmental effects *e_ik_* (which might include genetic effects originating from other species not controlled for here, see below). This is the basic rationale in community genetics: genetically related individuals belonging to a focal species (also termed “foundation species” in the ecological literature) will favor similar patterns and similar values for the community-level variable (Whitham et al., 2003).

The value of *c* is treated as a “community phenotype” or a community-level variable (Box 1): the phenotype of one or several associated species with a genetic variance $\sigma_{g}^{2}$ and “community heritability” $H_{c}^{2}=\sigma_{g}^{2}/\left(\sigma_{g}^{2}+\sigma_{e}^{2}\right)$ (Fritz and Price, 1988; Whitham et al., 2006). We use uppercase *H* to denote the broad sense heritability usually estimated in community genetics. Community genetics usually investigates the effect of the genetic variation in a focal plant species on its associated arthropod community. The experimental settings typically consist in a common garden with randomized replicated clones of the focal species and measurements taken at the community level such as arthropod abundance or species richness (reviews in Haloin and Strauss, 2008; Genung et al., 2011; Tack et al., 2012).

In such biological systems, the variable *c* only considers the IIGE on the arthropod community traits, excluding DGE on the plant species. Community heritability is therefore a measure of IIGE variance (which would not be true if *c* was, e.g., the total biomass of the whole plant-arthropod system). Thus, we now refer to IIGE heritability, $H_{I\,I\,G\,E}^{2}$. The notion of IIGE heritability as presented above presents an analogy with population-level heritability without assuming that communities can be selected as a whole (Collins, 2003). It is thus a matter of some debate. Could $H_{I\,I\,G\,E}^{2}$ be predictive of the response to selection of the community trait according to the breeder’s equation, i.e., $\Delta \,\bar{c}=H_{I\,I\,G\,E}^{2}\,S_{c}$ ? (with *S*_c_ a hypothetical selection differential on *c*). Community ecologists who quantified this parameter warned against such interpretation (Whitham et al., 2006; Genung et al., 2011). Whitham et al. (2006) asserted that this approach does not imply that communities have a fitness in the wild or evolve as populations do. Rather, they called for an interpretation of $H_{I\,I\,G\,E}^{2}$ as an integrative measure of the cascading effects of genes of the focal species at the community level. This means that $H_{I\,I\,G\,E}^{2}$ should be taken as a measure of an association to estimate how much of the variance in the community variable results from genetic variation in the focal species.

#### Practical application for breeding

**FIGURE 2:**
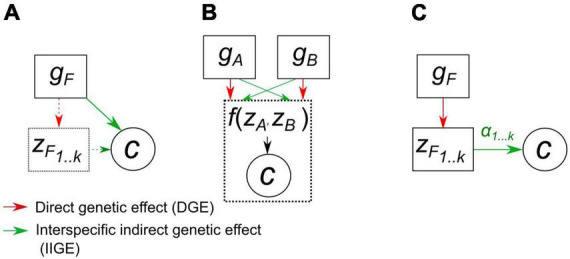
Causal pathways between genetic effects, *g*, and a community variable, *c*, as assumed in the quantitative genetic models described in the main text. **(A)** IIGE heritability approach: a genetic variance component of a focal species (*g*_*F*_) is used to quantify the genetic basis of variation in a community trait. The direct genetic effect on the focal species trait are ignored (dotted line). **(B)** Joint phenotype approach: the genetic variation of two species is treated symmetrically, considering for each their respective direct and indirect genetic effects. This is a variance component analysis that might either decompose on each species phenotype or directly on the community variable, the joint phenotype, if the two species are harvested together and not sorted. **(C)** Community-level index for traits: the community variable is regressed on the *l* traits of the focal species. The regression coefficients are then used to project genetic changes of these traits onto the community variable using a standard linear selection index approach.

The notion of IIGE heritability was designed to investigate the strength of IIGE of host plants on other taxa. To what extent could it be useful for breeders interested in improving plant communities? The first practical advantage over a more fine-grained approach (see below) is that it is trait free: it does not investigate the effect of the phenotype of the focal species on the community variable (Figure 2A) and instead quantifies IIGE as a whole, thus limiting the need for trait phenotyping. For estimating IIGE heritability, a breeder would first have to choose a focal species and control for its genetic variation within a standard range of experimental crop communities. $H_{\mathrm{IIGE}}^{2}$ would provide insights into the expected efficiency of selection of the focal species with respect to the improvement in the community variable.

The only example we found of $H_{I\,I\,G\,E}^{2}$ estimates in a crop community in the literature was provided by Maamouri et al. (2015). These authors grew 46 contrasted lucerne genotypes (*Medicago sativa*) with a grass (*Festuca arundinacea*) grown as a companion crop. While they found strong average broad-sense heritability for the direct genetic effect on lucerne biomass (*H*^2^ = 0.76, range across sampling rounds: [0.64 – 0.83]), the broad-sense heritability on the grass biomass was much lower ($H_{I\,I\,G\,E}^{2}$ = 0.05 [0.00 – 0.17]). This suggests that selection on lucerne biomass would have at most minor consequences for the grass biomass.

Heritability estimates of IIGE or at the community level are exposed to the same flaws as heritability estimates at the population level (Hansen et al., 2011), including high dependency on the study context and assumptions and computation preferences (Wilson, 2008; Firmat et al., 2017). When the community variable is on an arithmetic scale such as yield, this can be partly improved by computing the coefficient of genetic variation for IIGE (i.e., the IIGE standard deviation standardized by the mean value of *c*, refer to Hansen et al., 2011). Such mean standardized estimates could help to perform meaningful comparisons across breeding populations and testing sites to adjust community-level selection strategies.

Although in nature, the value of *c* is not directly associated with a value of fitness (genetically based selection does not act at the community level), this might be the case in a breeder’s field if artificial selection is performed among isolated and genetically controlled communities. In this case, $H_{I\,I\,G\,E}^{2}$ estimates might become interpretable within the breeders’ equation. However, DGE and IIGE can sometimes be selected as a whole. This is the situation addressed by the breeder’s version of the joint phenotype approach described in the following section.

### Approach 2: Joint phenotype

#### Generalities and theoretical grounding

Summing the genetic contributions of several species in a community trait is an alternative approach to interfacing responses to selection between the population and the community-level. The simplest is to sum the genotypic values of each interacting species (Queller, 2014):


(6) $$c_{i\,j\,k}=g_{A\,i}+g_{B\,j}+e_{i\,j\,k}$$

where *g*_*Ai*_ and *g*_*Bj*_ are the breeding values for individual *i* of species *A* and individual *j* of species *B*, respectively. According to this statistical expression, the joint phenotype *c* is defined by Queller and Strassmann (2018) as “a trait or outcome that can be affected, and potentially evolve, under the influence of two or more parties.” Both species are treated symmetrically and the joint phenotype results from the potential interaction between species, but the details of these interactions (values of interacting traits, complex feedbacks, etc.) are treated as a black box (Figure 2B). The evolutionary properties of joint phenotypes were investigated by Queller (2014) by applying the Robertson-Price equation (Frank, 2012). Queller (2014) showed that the change in *c* is predicted by the sum of the genetic variance for each species, respectively multiplied by their selective effects on *c*, i.e., the covariances σ(*w_*A*_, c*) and σ(*w_*B*_, c*). A non-zero covariance between fitness *w* and *c* indicates that natural selection acts on species’ traits that affect the community variable (Johnson et al., 2009). When the two covariances for each species are of opposite signs, a change in *c* results from an evolutionary conflict between the two species: an increase in fitness in one species is constrained by increased fitness in the other species.

If *c* is the amount of light captured within a plant community (Queller and Strassmann, 2018), an increase in size in species *A* will increase its competitive effect by negatively affecting the fitness of species *B*, and vice versa. The conflict can be resolved and *c* increased further if, for instance, one species evolves a strategy of shade tolerance, generating niche differentiation with respect to light. Recent studies in multispecies grasslands showed that niche differentiation and complementary resource use can evolve in a single generation (van Moorsel et al., 2018; Meilhac et al., 2020). As such, the concept of joint phenotype developed by Queller (2014) might provide a relevant theoretical framework to investigate the genetic determinants of such patterns. However, the analysis by Queller (2014) assumes regulation processes under natural selection in both species, which is of limited relevance for guiding artificial selection at the community level, which requires more specific modeling.

An analog version was coined for plant breeding many years ago. Wright (1985) proposed a selection model relying on the variance decomposition of a joint phenotype summing the “economic yield” of each species. This approach was inspired by factorial designs used to improve the parental population for breeding schemes for hybrid maize. The scale shift here relies on an analogy between the genetic combining ability of alleles (i.e., within a genome) from inbred lines and the ecological combining ability of genotypes (i.e., among distinct genomes) from different species.

#### Practical application for breeding

Wright (1985) approach aimed at modeling genetic gain on the joint phenotype when selection is performed at the scale of the population of bispecific communities that differ in their genotypic composition. Fully dissecting the genetic variation in the joint phenotype across a population of genetically controlled artificial communities requires a factorial design including a large number of combinations of each genotype of species *A* sown with each genotype of species *B*. Accordingly, two sorts of variance components of *c* can be partitioned: (1) the average or general effect of a genotype of one species on the joint phenotypes *c*, i.e., the general mixing ability (GMA) including both DGE and IIGE of this genotype on *c*; (2) the specific effect resulting from genotypic interactions deviating from additivity and quantifying the specific mixing ability (SMA) of individual pairs of genotypes on *c* (for more details see: Annicchiarico et al., 2019; Sampoux et al., 2020). Put simply, the GMA component of variance includes both additive direct genetic effects and effects of additive ecological interactions on *c* (average IIGE of a genotype across the factorial), whereas the SMA component of variance quantifies the contribution of non-additive effects on *c.*

Today, 35 years after Wright’s publication, no prediction based on a full decomposition of the variance of *c* in a bispecific community has yet been made. This is likely due to the demanding experimental design required for such a decomposition. For *two* species and a minimum of 30 candidate genotypes each and three replicates per pair, the number of plots would be 3 × 30^2^ = *2,700*, corresponding to a huge single site experimental design beyond the reach of most breeding programs. With a third species, the required experiment becomes completely unrealistic (81,000 plots) (but refer to Haug et al., 2021).

Specifically targeting the GMA variance of interacting species (Hill, 1990) can substantially reduce experimental requirements. This involves parallel selection of the GMA of the two species, each with a specific (recurrent) selection process. For each of the two species, candidate genotypes of the selected species (let’s say species *A*) are sown in mixture with a mixture of representative genotypes from the other (unselected) species (let’s say species *B*), used in the mixture as a tester for genotypes of *A*. In parallel, the same procedure is performed for species *B* with a mixture of representative genotypes of species *A* used as testers (i.e., selection in parallel for GMA, refer to Figure 2 in Sampoux et al., 2020). According to Sampoux et al. (2020), when targeting GMA variance for *c* by selecting on, e.g., species *A*, the measurable component of the joint phenotype *c* is as follows:


(7) $$c_{A\,i\,j}=\mu_{A}+\mu_{B}+v_{A\,i}+a_{A\,i}+{}_{A\,i\,j}$$

with μ*A* and μ*B* the mean value of species *A* and *B* contributing to the community value *c*. Here, the controlled genetic variance of *c* in the design is only due to species A, with *v*Ai** and *a*Ai** the direct genetic (DGE) and the indirect genetic effects (i.e., IIGE, *a* for “associated effect” in breeding terms) of species *A*, respectively. Together, these terms model the GMA of the *i*th genotype of species *A*. From this expression, improving *c* by selecting on species *A* only gives the following expected response for the joint phenotype:


(8) $$\Delta \,\bar{c}=\frac{{\bar{i}}_{\text{c}}}{\sigma_{\text{c}}}\,\left[\sigma_{v_{\text{A}}}^{2}+2\,\sigma_{v_{\text{A}},a_{\text{A}}}+\sigma_{a_{A}}^{2}\right]$$

${\bar{i}}_{c}$ is the selection intensity on the value of *c* and σ_*c*_ the total phenotypic variance for *c*. Under the procedure of selection in parallel for GMA described above, the analogous expression can be derived for species *B* (with *A* as a tester), also contributing to genetic gain for *c*. The value between brackets is the variance of the joint phenotype caused by genetic variation only in species *A* on which selection is performed. This variance expression is the sum of two components: the direct response to selection on *c* for the contribution of species *A* ($\sigma_{v_{A}}^{2}+\sigma_{v_{A},a_{A}}$) and the correlated response on the value of species *B* ($\sigma_{v_{A},a_{A}}+\sigma_{a_{A}}^{2}$) [for a detailed argument including a release from the assumption of the equal species weights, refer to Sampoux et al. (2020)]. Both components include the covariance between the DGE and the IIGE of the selected species. Interestingly, eq. 8 parallels the results obtained by Griffing (1967) for the response to group selection at the intraspecific level, when assuming unrelated interacting individuals.

The main advantage of this joint phenotype approach is that it emphasizes the constraining role of this covariance term for community-scale genetic improvement. If σ_*v*_*A*_,*a*_*A*__ < 0, improving the contribution of species *A* (DGE) to the mixture while not accounting for its IIGE on *B* (*a*_*A*_) is not possible without weakening the contribution to the mixture of species *B* (Wright, 1985). This typically happens when selection for yield in *A* increases its competitive effect, thus weakening the contribution of *B*. Breeders that has to cope with an evolutionary conflict (*sensu* Queller, 2014) and manage a trade-off between species vs. community performance. Performing two selection processes in parallel (one for each species), as proposed by Sampoux et al. (2020) and described above, could be a promising way to deal with such an evolutionary conflict.

By allowing integrative decomposition of the genetic interactions underlying the variation in a community variable, the joint phenotype approach provides quantitative genetic expression of the among-species conflicts the plant breeder will have to deal with. However, this approach enables prediction across biological scales (from species quantitative traits to *c*) through estimates of covariances (i.e., σ_*v*_*A*_,*a*_*A*__) between traits measured in two different species (as the IIGE of species A affects, e.g., the yield of species *B*). The nature of this parameter exposes quantitative predictions to strong limitations of both biological and methodological origins, as we will see below (refer to section headed “Common limitations and links between the three approaches”).

### Approach 3: Community-trait genetic gradient

#### Generalities and theoretical grounding

In contrast to the two previous approaches, this approach relies on the functional trait assumption: a set of functional traits, and not genotypes, affects the community variable (Figure 2C). Investigating how a set of traits measured in a single species affects community functioning requires a multivariate approach linking the evolution of genetically correlated functional traits to a community-level phenotype. The multivariate selection model proposed by Johnson et al. (2009) relies on the notion of a “community-trait gradient”: a multiple regression of a single community trait *c* on a set of *l* evolving traits for a focal species. Variation in the community variable is therefore modeled as follows:


(9) $$c_{i\,k}=\mu_{c}+\sum_{j=1}^{l}\alpha_{j}\,g_{i\,j}+e_{i\,k}$$

where α_*j*_ is the partial regression coefficients for the *j^th^* trait and *g*_*ij*_ its genetic value for the *i^th^* genotype at trait *j*. The estimated vector of coefficients makes it possible to project the multivariate evolutionary change in a focal species on the ecological variable. This is a way of modeling the IIGE of a complex, multivariate phenotype on a predefined community variable. This “matrix projection model” therefore builds on Lande’s (1979) formulation of the breeder’s equation to extend it to a community variable (Johnson et al., 2009):


$$\Delta \,\bar{c}=\left[\alpha_{1},\alpha_{2},\ldots ,\alpha_{l}\right]\,\left[\begin{array}{cccc} G_{11} & G_{12} & \ldots & G_{1\,l}\\ G_{21} & G_{22} & \ldots & G_{2\,l}\\ \vdots & \vdots & & \vdots \\ G_{l\,1} & G_{l\,2} & \ldots & G_{l\,l} \end{array}\right]\,\left[\begin{array}{c} \beta_{1}\\ \beta_{2}\\ \vdots \\ \beta_{l} \end{array}\right]\,\left(10\,a\right)$$

or, for a more synthetic notation:


$$\Delta \,\bar{c}=\alpha^{T}\,\text{G}\,\beta \;\left(10\,b\right)$$

The second and last terms are the elements of the standard multivariate breeder’s equation, respectively, the G-matrix, and the linear gradient of selection (Lande and Arnold, 1983), i.e., the vector of partial regression coefficients of each *j* trait on fitness, whose product gives the expected response to selection in the vector of traits. The first term is the vector of partial regression coefficients (estimated independently in eq. 9), allowing the projection the genetic changes on the community variable (the subscript *T* denotes the transpose of this vector). This projection of evolutionary change corresponds to the sum of the evolutionary changes for the *l* traits, weighted by the α*j* coefficients (refer to Appendix 1, for a derivation of this expression using the Robertson theorem). In ecological terms, these coefficients represent the level to which each *j* trait is “functional” (refer to Litrico and Violle, 2015), i.e., interact with the species of the targeted community and affect the community-level variable.

Johnson et al. (2009) originally used this model to investigate the effect of functional trait variation in a wild species *Oenothera biennis* (Onagraceae) on the associated arthropod community. The model described above helped these authors formalize a set of conditions for the evolution of IIGE on the focal plant species: the trait *j* should be genetically variable (*G*_*jj*_ > 0), selection should affect the trait (β_*j*_ ≠ 0), and the trait should cause variation in the community variable (α_*j*_ ≠ 0).

#### Practical application for breeding

Johnson et al. (2009)’s approach is strictly analogous to a linear selection index: it weights the sum of change in each dimension of a multivariate phenotypes to project it on a single variable, i.e., the selection index. The only – but nevertheless significant – difference is that the coefficients of the index are estimated statistically (eq. 9, refer to Appendix 1). Selection indexes are widely used by breeders and their properties are well known (Hazel et al., 1994). According to the properties of the linear selection index (Lin and Allaire, 1977; Nordskog, 1978) and using Johnson et al. (2009)’s notation, the heritability of the community variable described by *k* underlying traits is given by:


(11) $$h_{c}^{2}=\frac{\alpha^{T}\,\text{G}\,\alpha }{\alpha^{T}\,\text{P}\,\alpha },$$

with **P** the phenotypic variance–covariance matrix among traits. Thus, the response to selection in equation (10b) can be expressed in the selection intensity form, which is more familiar to breeders:


(12) $$\Delta \,\bar{c}={\bar{i}}_{c}\,\frac{\alpha^{T}\,\text{G}\,\alpha }{\sqrt{\alpha^{T}\,\text{P}\,\alpha }},$$

with ${\bar{i}}_{c}$ the univariate intensity of selection applied to the community variable.

From a practical point of view, this approach includes two independent steps. The first step aims to estimate how the phenotypic trait values of the focal species affect the community variable (i.e., estimating α). The second step aims to quantify the genetic architecture of the ecologically relevant traits previously identified (i.e., estimating **G**).

This approach has two potential advantages. First, by explicitly modeling ecological functions in the first step, it avoids the potentially problematic use of covariances between IIGE and DGE, which play a central role in joint phenotype approaches, as this covariance is suspected of strong variability among experimental contexts (discussed in the following section). Second, the main practical advantage of this trait-centered approach is that it makes it possible to discard traits that have no influence on the target community variable *c* and to focus on the traits that have the main effects. From a multiple traits-community variable regression (eq. 9), a standard model selection procedure using information theoretical approaches (review in Grueber et al., 2011) could be used in the first step to discard traits that do not deserve to be taken into account for the improvement of *c*. This would make it possible to considerably reduce the size of the sample required for further quantitative genetic predictions in the following prediction step (eq. 10). Estimates of α could rely on biological and experimental knowledge from functional (agro)ecology (Garnier and Navas, 2012). The analysis of α would provide an innovative way to design for the focal species what we call “community-ideotypes,” i.e., ideotypes that not only target the focal species’ own performance but also the expected performance generated through IIGE by such an ideotype at the scale of the community. However, one should keep in mind that a trait *j* with no community-level effect (α_*j*_ ≈ 0) may still be useful to define the selection index due to its genetic correlation with other ecologically relevant traits.

Another key advantage of this approach is that it allows breeders to map the consequences of genetic constraints (i.e., genetic covariances among the focal species’ traits) in terms of response at the community scale. Let us assume a simple situation where the genetic covariance among two traits is positive (*G*_12_ > 0) and both α_1_ and α_2_ for these traits are also positive. Then, selecting for trait 1 alone is expected to improve the contribution of both traits to the community performance (i.e., as $\Delta \,\bar{c}=\alpha_{1}\,G_{11}\,\beta_{1}+\alpha_{2}\,G_{12}\,\beta_{1}$). But if α_1_ and α_2_ are of opposite signs (α_1_ > 0, denoting, e.g., a direct genetic effect on total yield and α_2_ < 0, denoting, e.g., a negative competitive effect on a companion legume that supplies nitrogen), selecting for trait 1 alone is expected to reduce the value of trait 2 due to the positive genetic covariance. In other words, this could enable breeders to easily objectivize the consequences of their usual (i.e., species-centered) work at the community scale, by generating anticipatory predictions at this level. For instance, by extending the standard breeding indices based on a series of traits measured in a pure stand to predict performance in a mixed stand (e.g., Annicchiarico, 2003), this approach would make it possible to predict the consequences of this species-specific performance at the community scale. This includes the quantitative genetic exploration of the promising notion of “biological interaction function” coined by Haug et al. (2021). To sum up, Haug et al. (2021) suggest correlating both direct and indirect genetic effects with measured traits that generate different types of species interactions in the mixture, leading to the identification of traits that produce favorable biological interactions at the community scale. To identify suitable cultivars for cereal–legume mixtures, Kammoun et al. (2021) argue for a simple statistical approach that predicts reliable mixtures based on pure stand performances for both components combined with a description of their interaction function. We believe that such a basic set of variables would be a reliable starting point for designing selection criteria for this type of simple crop mixture.

This approach is based on the predictions of *c* from genetic (co)variation in a single species. It therefore assumes that one species needs to receive more attention than any other species from the breeder. We observed that this is the most frequently reported case in the plant breeding literature (for recent examples: Annicchiarico et al., 2021; Ergon and Bakken, 2022; Moutier et al., 2022).

### Common limitations and links between the three approaches

#### Integrative expressions for community-level selection response

The three approaches we identified are distinct in several conceptual, statistical, and practical dimensions (Table 1). These approaches rely on distinct strategies for modeling causal pathways between genetic variation and community-level variation (Figure 2). However, a general model linking all three approaches is possible. The community-trait genetic gradient approach could theoretically be extended to more than one species. The evolution of *c* affected by two species *A* and *B* represented by *l* traits each (kept equal for the sake of simplicity) can also be modeled as the evolution of a linear index:


(13) $$\Delta \,\bar{c}=\sum_{j\,\left(A\right)=1}^{l}\alpha_{A\,j}\,\Delta \,{\bar{z}}_{A_{j}}+\sum_{j\,\left(B\right)=1}^{l}\alpha_{B\,j}\,\Delta \,{\bar{z}}_{B_{j}}$$

**TABLE 1:** Main features of the three modeling approaches described in the main text.

Modeling approaches:	(1) Heritability of IIGE	(2) Joint phenotype	(3) Community-trait genetic gradient
Analogy/similarity with standard breeding concepts	Heritability	Factorial designs for hybrid maize breeding schemes	Linear selection index
Focal species-centered	Yes	No	Yes
Trait-based	No	No	Yes
Application to multivariate phenotypes	By dimensional reduction	Difficult	Designed intentionally
Approach for partitioning genetic variance of *c*	Components of variance	Components of variance	Regression on species phenotypic traits associated with an estimate of the genetic (co)variances of the trait.
Key informative parameter for breeding	Strength of IIGE for *c* ($\sigma_{a_{B}}^{2}$ or $\sigma_{I\,I\,G\,E_{A}}^{2}$ for species B cf. eq. 5)	Sign of the genetic covariance between DGE and IIGE (σ_*v*_*A*_,*a*_*A*__ or σ_*DGE*_*A*_,*IIGE*_*A*__, i.e., the genetic covariance of traits measured among interacting species)	Sign of the product between the expected genetic change for trait *j* and its community-scale effect ($\alpha_{j}\,\Delta \,{\bar{z}}_{j}$)

**FIGURE 3:**
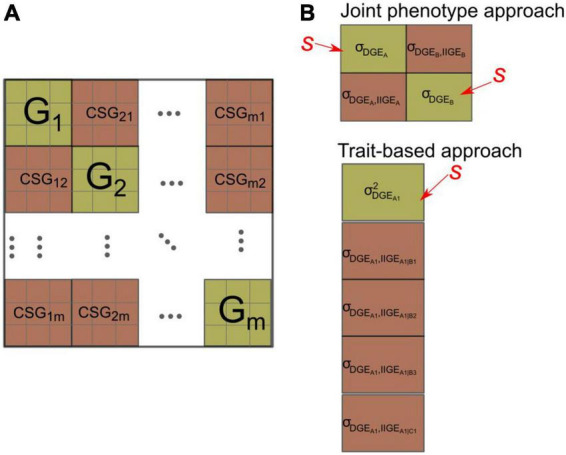
**(A)** Structure of the compounded community-level G-matrix that would be required to be able to predict genetic changes in species traits in a community of *m* species potentially under selection for their DGE. It includes both G-matrices (in green) on the diagonal (G_1_, G_2_… G_*m*_) for each species (equivalent to the one in eq. 10a) and reciprocal cross species G-matrices (CSG) (in dark red), representing genetic covariances among species traits, i.e., covariances between DGE and IIGE for each pair of traits belonging to different species. The figure depicts three measured traits per species. See main text for a discussion on the limits of the concept of the compounded community-level G-matrix in relation to DGE and IIGE. **(B)** Same representations for specific examples of the CSG-matrices that would be required to predict the response to artificial selection at the community level [with “s” indicating the trait(s) under selection]. Top: The particular case of the joint phenotype approach with selection on the DGE of each species. The prediction of the community variable c then requires the variance of DGE for each species and the covariances between DGE and IIGE affecting the other species. Bottom: Example of a trait-based approach with selection only applied to trait *A*_1_ of species *A*, and predictions focused on three traits of species *B* (*B*_1_–*B*_3_) one trait of species *C* (*C*_1_), all being potential components of the community-level target c (refer to main text for details). In cases where the magnitude of the genetic covariance between DGE and IIGE is non-null, the evolution of this first trait is expected to affect the magnitude of IIGE on other species’ traits, hence the mean value of other species traits. The notation σ_*DGE_A1,IIGGE_A1—B1*_, for instance, denotes the covariance between the DGE of species *A* associated with trait *A*_1_ vs. its IIGE affecting the trait *B*_1_ of species *B*.

This equation simply sums the ecologically weighted effects of each evolving trait for each species. But note that changes in traits in species *A* and *B* are not independent, but are linked through genetic covariance across each pair of species traits, caused by genetic interactions among species (IIGE) mediated by interaction traits (*sensu* Litrico and Violle, 2015). The sum of the trait’s specific α for each species corresponds to their respective weights regarding their contribution to *c*. The generalization of equation (13) for *m* interacting species with *l* traits each is:


(14) $$\Delta \,\bar{c}=\sum_{i=1}^{m}\sum_{j=1}^{l}\alpha_{j\,\left(i\right)}\,\Delta \,{\bar{z}}_{i_{j}}$$

with the first and second sigma summing effects over species and over traits, respectively. Interestingly, obvious links exist between this general expression and the integrative functional parameters used to describe the trait-based diversity of functional types in a plant community (refer to Violle et al., 2007; Garnier and Navas, 2012). The issue, however, is that full predictions of selection response for such an index require a complex set of interacting parameters involving both species-specific G-matrices and reciprocal cross-species genetic covariance matrices (CSG), describing the interspecific, among trait genetic relations between DGE and IIGE between pairs of species (Figure 3). To our knowledge, the only estimate for such a combined G-CSG-matrix, i.e., including both the direct and indirect genetic effects for a focal species, was provided by Riedel et al. (2018). However, these authors did not report estimate uncertainties and did not compute the effect on an integrative, index-like, community-level variable.

A CSG-matrix combines the covariance between the DGE of a given species *A* with its IIGE (originating from *A*) on the partner species’ traits. It thus involves the association between traits measured in two different species (e.g., a biomass trait measured in a forage crop species *A* [DGE], that through IIGE, affects a grain yield trait measured in an associated cash crop species *B*), i.e., in totally unrelated individuals. This type of covariances is therefore distinct from standard genetic covariances among traits measured in the same series of individuals as well as for DGE and IGE measured in interacting individuals of the same species (Griffing, 1967). These genetic covariances among species’ traits are complex parameters that include both genetic and ecological (i.e., species interactions) causal pathways. They are pivotal parameters in the joint phenotype approach (Wright, 1985; Sampoux et al., 2020) and in some versions of multitrait approaches to community genetics (Riedel et al., 2018) as they make it possible to link population genetics to the community scale.

If the capacity of the general equation cited above (eq. 14) to provide reliable predictions should be considered with great caution, it is nevertheless useful for clarifying the statistical and conceptual links between the three approaches described above. With *l* measured traits in a single species (*m* = 1) eq. (14) reduced to Johnson et al.’s. (2009) community-trait gradient approach (eqs. 10a, 10b, and 12). With two species (*m* = 2), each with a single measured trait (*l* = 1) and no estimated ecological gradient (α_*A*_ = α_*B*_ = 1), this becomes Wright’s (1985) style joint phenotype approach (eq. 8). If the latter assumption is further reduced to one species, then the last remaining parameter is a variance component analogous to IIGE *H*^2^ approaches. This is also equivalent to a joint phenotype approach with only the IIGE effect measured (i.e., in eq. 6, only $\sigma_{a_{A}}^{2}$ is assumed to be non-zero), underlining the fact that community *H*^2^ approaches are a special case of the joint phenotype, with neglected direct effects. Breeding based on the community *H*^2^ approach would then be possible if the community is taken as the unit of selection (i.e., the breeder assigns a fitness value – whether or not the genotype is selected – to the community-level performance of a genotype).

### Cross-species genetic correlations and inaccurate quantitative predictions

It will be recalled that when restricted to traits measured in the same species, genetic correlations (a standard measure for genetic covariances) are often the poor predictors of the actual correlated response to selection (Gromko, 1995), and obtaining reliable predictions of the correlated response to recurrent artificial selection in plants seems very challenging (Pélabon et al., 2021). It has also been shown that estimates of species-specific G-matrices are environment-dependent (Wood and Brodie, 2015), i.e., the environment in which they are measured influences their estimation, which can introduce a strong bias in predictions that were made for another environment. Taken together, what precedes might explain why below a few hundred measured individuals, it seems reasonable to draw qualitative instead of quantitative conclusions (Lynch and Walsh, 1998), that is, to determine whether correlations are significantly negative, positive, or non-different from zero.

These limitations are expected to be even stronger for the covariances between DGE and IIGE, by definition measured on traits from two ecologically interacting species. First, the IIGE includes ecological interactions that are not explicitly accounted for in quantitative genetics models. This may introduce a strong bias resulting from external environmental factors that affect both species. Second, the evaluation and selection of genotypes within experimental plant communities typically requires reducing intra-plot genetic diversity for the selected species to the single genotype under evaluation. It has been shown that when genetic diversity is low, the traits of bi-specific experimental communities are more strongly exposed to experimental stochasticity and are hence less reliable (Milcu et al., 2018). Third, in certain experimental cases (e.g., in perennials), natural selection may co-select genotypes among species (van Moorsel et al., 2018). If not accounted for, this could bias the level of genetic covariance between DGE and IIGE due to processes analogous to linkage disequilibrium (cf. Wade, 2003).

Consequently, as it includes ecological causalities, the genetic (co)variances among species’ traits (the elements of CSG) are not of the same nature as species-specific covariances (the triangle elements of G-matrices) and are not exposed to exactly the same sources of bias. Thus, they should neither evolve nor drive the evolution of *c* according to the same processes. We suggest that this is the main limitation of the biological scale-coupling approaches from a quantitative genetic viewpoint. To sum up, accurately predicting selection response at the community scale might be challenging as prediction is contextual at this level. This suggests that breeders looking for anticipatory predictions will have to choose the “least bad” assumptions for their breeding context and targets.

## Future directions

Our analysis of extant models underlines the fact that predicting the effects of artificial selection performed simultaneously on more than one species is technically challenging. This is first due to the size of the factorial experimental designs required (but refer to suggestions from Haug et al., 2021), especially if several functional traits need to be measured in each species. However, the need for estimates of genetic covariances between traits measured both within and among species (for IIGE) is a further obstacle. Reliable parameter estimations (e.g., for genomic selection) might therefore require an unusually large number of test environments (refer to Annicchiarico et al., 2021).

### Mobilizing the modeling approaches for community-level breeding strategies

The complexity of mixed ecological and genetic systems reminds us that these different modeling approaches should probably not be merged into an ideal, all-purpose integrative modeling approach. Instead, we suggest that they should serve as an adaptable toolkit for breeders to obtain the anticipatory predictions they need. We now present some situations and strategies in which these modeling approaches could be used.

A frequent pattern observed in plant communities is that all species do not contribute equally to community-level performance (Mahaut et al., 2020), which is, as pointed above, often assumed in the plant breeding literature for mixture. The respective contribution of each species is contextual and depends on both the agronomic context and the breeding objectives (Box 2). Once the species composition of the target community to be improved is established, a reasonable breeding strategy should start by identifying the key species to breed as a priority to achieve the highest possible community-level genetic gain with the minimum breeding effort (note that the relevance of the focal species might not be determined by relative biomass of each species in the cover). Such focal species should be economically relevant and/or should have a major ecological effect on community performance, and the genetic variance in the indirect genetic effects should be strong (typically $H_{I\,I\,G\,E}^{2}>0.5$). The breeding potential of this species could be identified through gross estimates of IIGE heritability (“Approach 1” above) for the available genetic resources and within the targeted community.

If genetic improvement requires the simultaneous selection of several species (i.e., if the breeder assumes there is no obvious single “focal species” to reach a breeding goal), the joint phenotype approach (“Approach 2”) could be used to identify the strength of negative correlations between DGE and IIGE and to select the appropriate breeding scheme to overcome these constraints (Sampoux et al., 2020). This strategy then resembles the intra-specific breeding context where the magnitude of the negative (hence constraining) correlation between DGE (i.e., individual genotype performance) and IGE is estimated and accounted for (e.g., at the population level : Costa e Silva et al., 2013).

If a focal species has been clearly identified, one possible strategy is to identify the key traits of this focal species for performance at the community level. The community gradient approach of Johnson et al. (2009) described above (“Approach 3”) appears to be a suitable integrative theoretical framework for this purpose. In practical terms, the goal was to identify the traits of the focal species with both: (1) high absolute values for their α coefficients (eq. 10a), i.e., with strong (or non-negative) effects on the community-level performance and (2) substantial standing genetic variation in the pool of available breeding candidates. However, the limitation of this trait-based approach is that it does not account for reciprocal interactions with other species potentially under selection. Reciprocal interactions may bias estimates of the partial regression coefficients α, as shown with the interaction coefficient of models for intra-specific genetic effects (Bijma, 2014). This could be overcome by coupling this quantitative genetic model with recently developed of ecophysiological models of crop mixtures (Louarn et al., 2020). These models could be adapted to provide the values of the α coefficients for a given context and with respect to a given breeding target, while accounting for reciprocal species interactions (for further discussion on this topic, see Bourke et al., 2021 in this special issue).

If the reciprocal interactions are shown to have negligible effects on the selection process, this trait-based approach is similar to basic selection index theory extended to the community scale and could be readily implemented by breeders (e.g., leading to the design of “community level ideotypes” for the focal species). Conversely, under strong reciprocal interactions, the best pair(s) of the few best performing genotypes identified in the previous selection efforts could be finally selected using a full factorial design of reasonable size, with a limited number of genotype entries (e.g., design of Moutier et al., 2022), which makes it possible to account for both GMA and SMA (cf. Wright, 1985) (however, note that there is no clear evidence for the importance of SMA variance in the experimental literature; Annicchiarico et al., 2019). Such an optional final selection step could be particularly useful if fine-tuned co-adaptation of genotypes is required (i.e., requiring well-defined and stable growing conditions, which might not be much frequent in a low input agroecological future).

### The realized response to selection: Assessing the efficiency of modern breeding for crop mixtures

Assessing and predicting the efficiency of current breeding practices is of major concern to adapt breeding schemes to agroecological objectives. Breeding efforts in the recent decades aimed at pure stand performance might retain some efficiencies for the focal species in mixtures (Annicchiarico et al., 2019). However, intensive selection for monocropping performance that has led to current elite varieties is suspected to have reduced the relevant genetic variation for important traits at the community level, with potentially negative IIGE resulting from overcompetitive genotypes.

Several common garden experiments comparing cultivars according to their registration years have documented important genetic gains for monocropping yield traits (e.g., Sampoux et al., 2011; Laidig et al., 2014; Rose and Kage, 2019; Voss-Fels et al., 2019; Herrera et al., 2020). Although some of these studies investigated the effects of management conditions (e.g., low vs. high nitrogen fertilization, conventional vs. organic management) on historical genetic trends, to our knowledge, none estimated the extent to which pure stand breeding in a focal species has affected community-level performance. Thus, we do not know the long-term effects of modern, monoculture-oriented plant breeding on the performance of crop mixtures.

Filling this knowledge gap would require a randomized experimental design comprising dozens of registered varieties (with known registration years) for a given focal species, each sown in a pure stand (experimental control) and in a mixture (experimental treatment). This would make it possible to measure (1) how genetic gain in pure stands is sustained in mixtures (direct genetic effect) as well as (2) the effects of the focal species on the community performance, i.e., IIGE on yield or yield stability. Cereal–legume intercrops or forage legume–grass mixtures would be the ideal candidate systems for such a test.

Estimating the realized selection response of the target species would be the ultimate test of the relevance of current breeding strategies for agroecological practices and their effect on plant–plant interactions. Having the realized selection response to selection on a single focal species for a community-level variable would exceptionally corroborate anticipatory predictions (Maris et al., 2018) useful for breeding [e.g., the parameters of Johnson et al.’s. (2009) model]. In addition, comparing genetic gain in a pure stand and in crop mixtures would help to identify cultivars and design ideotypes that combine both species-level performance and good ecological abilities. The community gradient approach proposed by Johnson et al. (2009) is a relevant framework for interpreting the results of such an experiment. Community-scale performance could be formalized in terms of interaction traits (sensus Litrico and Violle, 2015) by estimating the magnitude of their respective α coefficients.

### Investigating a blind spot: Genetic variation in the sensitivity to species interaction

Joint phenotype approaches assess genetic variation in mixtures for two or more species. The resulting statistical concepts such as GMA (Wright, 1985) make it possible to avoid distinguishing between the direct genetic effect of a species and its sensitivity to the IIGE of a companion species: its performance corresponds to its (experimental average for GMA) expression within a mixture of species, as measured in the experimental design. However, to date, most experimental breeding approaches used for mixtures have been focused on a single focal species in which genetic variation in assessed (review in Annicchiarico et al., 2019), likely due to the complex implementation of full factorial designs. Such approaches assume that IIGE variation only results from variation in the interaction trait of the focal species, i.e., all genotypes of the associated species affected by the IIGE react in the same way to the influence of the selected species. In the model proposed by Johnson et al. (2009), for instance, this is reflected in the fact that the α*i* coefficients (eq. 10a) are constants, a feature inherited from pioneering models on intraspecific indirect genetic effects (Moore et al., 1997). *A priori*, this assumption only holds if the IIGE of the focal species is estimated against a single genotype of the associated species (i.e., there is no genetic variation in the companion species, thus no genetic variation to its sensitivity to the effect of the focal species). If the focal species interacts with several associated genotypes, they may be more or less sensitive or robust to this interaction and a relevant source of variation in IIGE would be ignored in this case.

**FIGURE 4:**
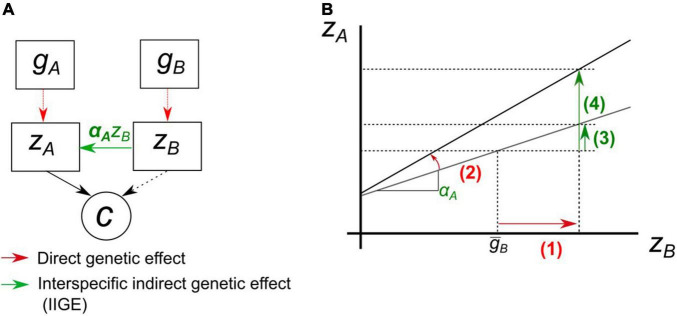
Schematic representation of the reaction norm approach for a focal species *A* with an agronomic trait, *z*_*A*_, regressed on the interaction trait of a second species *B*, *z*_*B*_. **(A)** Each species is influenced by its own genotype. *z*_*A*_ deviates under the influence of *z*_*B*_ times its sensitivity to this trait α_*A*_, i.e., slope of the reaction norm to *z*_*B*_ effect. The slope α_*A*_ is itself a trait of species *A*, can be genetically variable and respond to selection. Both traits can potentially contribute to a community variable *c*. **(B)** Graphical representation of the path diagram. Selection on *z*_*B*_ (1) involves a change in *z*_*A*_ (3) through IIGE at α_*A*_ constant. With the same amount of change in the interaction trait *z*B**, if selection now also targets the genetic value of the slope (2), the magnitude of the IIGE is modified, here increased (4).

This variation can be modeled by assuming that **α** coefficients (in eq. 10a) are genetically variable. The graphical model in Figure 4 illustrates how the distinction made by Litrico and Violle (2015) between agronomic and interaction traits can be made operational to explore such an assumption (refer to Navas and Violle, 2009). Instead of estimating species interaction as a factorial design combining individual genotypes, a trait of major agronomic value for species *A* can be regressed against a trait of species *B* involved in species interaction (e.g., biomass or an estimate of competitive effects). Assuming that the sensitivity of *A* to this interaction is genetically variable makes it possible to investigate specific genetic effects in a crop mixture (refer to section headed “Approach 2” above), while avoiding the need for a huge factorial design.

We suggest that the sensitivity of *A* can be modeled using a reaction norm approach, taking the value of the interaction trait of *B* as the environmental predictors for the agronomic trait of *A*. The properties of reaction norms under artificial selection have been well modeled (Kolmodin and Bijma, 2004). Modern statistical tools and concepts to analyze the genetics of reaction norms in plants, such as genetically informed random-regression models (Arnold et al., 2019), could easily be extended to interspecific interactions. This would make it possible to compute the genetic variance for the slope *G*(α_*A*_) and its genetic covariance with the genetic value of the agronomic trait *G*(α_*A*_,*g*_*A*_). As the ecological interaction is modeled by a genetic-by-functional trait(s) interaction, this strategy would help to avoid the huge factorial experiments (Wright, 1985) required to estimate IIGE variance when the species genetic background interacts. In situations where the slope of the reaction norm is genetically variable, breeding for increasing (or decreasing, if IIGE is dominated by, for instance, negative competitive effect) the value of this slope could be a relevant target to achieve breeding goals by acting on the “leverage effect” of the slope illustrated in Figure 4B.

## Conclusion

It is now clear that major chapters of quantitative genetic theory must be adapted to align current plant breeding efforts with increasing sustainability challenges. Quantitative genetics provided plant breeders with a scientific framework depicting the complex systems they needed to transform by both managing genetic variation and selection intensity. In the future, plant breeders will have to manage the consequences of their efforts for the improved functioning of complex crop communities. While breeding and evolutionary ecology evolved from the same theoretical background (Bijma, 2020), most relevant models to link the population to the community level of biological diversity have been designed in evolutionary ecology. Our review stresses that inputs from evo-to-eco models have the potential to shed light on the relevant properties of this scale shift to guide the development of future breeding activities. We now conclude by underlining the main practical implications of our survey:

(1) Identifying species with major effects on the breeding target will facilitate the prediction procedure. However, when the total number of species to be considered at once increases, quantitative genetic modeling will quickly reach its limits in providing meaningful anticipatory predictions, as the system becomes poorly controllable by artificial selection.
(2) Context dependence is expected to increase with diversity, rendering any anticipatory prediction of the response to artificial selection extremely unreliable. Furthermore, for such complex systems, empirical approaches based on evolutionary processes might be more cost-effective (Annicchiarico et al., 2019) and could represent valuable “stopgaps” (Hill, 1996) in the absence of efficient breeding schemes that can be implemented at the community level. The models we reviewed here could serve as baselines for ex-post interpretation of these empirical results and help to made them more reproducible in practice.
(3) Each of the three types of models we identified can be treated as a particular case of a multispecies-multitrait selection index approach. However, shifting the scale of quantitative genetics toward community performance involves dealing with genetic covariance among traits measured both within (DGE vs. DGE) and among species (DGE vs. IIGE), which have an intrinsically distinct biological nature. They consequently evolve and constrain community-level genetic change according to different processes that are not fully accounted for in the estimation procedure, which could finally drive anticipatory predictions away from reality.

We have provided a first synthesis of extant models linking quantitative genetics to community variation, identified knowledge gaps and inherent limitations, and suggested some directions for future research. We focused on the operationality of the modeling approaches to provide anticipatory predictions (Maris et al., 2018) for breeders. This review emphasizes the potential complexity of fine-grained interactions among genotypes of different crop species. Coupled with the generally low predictability of short-term selection response in plants (Pélabon et al., 2021), this should encourage the search for robust qualitative evidence to facilitate the choice of breeding strategies. The overview we provided (summarized in Table 1) of the available models and their parameters should be useful in this regard. Such models could be incorporated in the framework of the complex experimental pipelines currently being developed to articulate farmers’ field experiments and plant breeding stations (Wolfe et al., 2021). This would facilitate the critical assessment and monitoring of such large-scale strategies in the future. More generally, plant breeders, possibly in interaction with evolutionary ecologists, could use this theoretical framework to design appropriate experimental settings and community-level breeding strategies. We have no doubt that this scenario has the potential to improve plant breeding practice to cope with current agroecological challenges.

## Author contributions

CF and IL provided the original idea. CF did the literature search, framed and wrote the manuscript with inputs from IL. Both authors contributed to the article and approved the submitted version.

## Appendix

### Appendix 1 | Using selection theory to recover the extended breeder’s equation proposed by Johnson et al. (2009) for a community variable

Johnson et al. (2009) provided a theoretical formulation to extend the breeder’s equation to a community variable, with selection affecting *l* traits of a focal species. The authors did not provide a formal derivation. As we will show, this equation can be derived from the standard multiple regression of traits on fitness on the one hand, i.e., the definition of the multivariate linear selection differential describing the individual fitness (Lande, 1979; Lande and Arnold, 1983):

$$w=\mu_{w}+\sum_{j=1}^{l}\beta_{j}\,z_{j}+e_{w}$$

And, on the other hand, the “evo-to-eco” multiple regression of the community variable *c* on the population traits:

$$c=\mu_{c}+\sum_{j=1}^{l}\alpha_{j}\,z_{j}+e_{c}$$

The response to the selection of traits on the community variable can be derived from the Robertson (1968) secondary theorem of natural selection (refer to Walsh and Lynch, 2018), i.e., by developing $\Delta \,\bar{c}=c\,o\,v_{A}\,\left(w,c\right)$. For the sake of clarity, we keep the linear notation, as follows:

$$\Delta \bar{c}=cov(\beta_{1}g_{1}+\beta_{2}g_{2}+\ldots +\beta_{l}g_{l},$$

$$\alpha_{1}g_{1}+\alpha_{2}g_{2}+\ldots +\alpha_{l}g_{l})$$

Then,

$$\Delta \,\bar{c}=\alpha_{1}\,\beta_{1}\,G_{11}+\alpha_{2}\,\beta_{2}\,G_{22}+\ldots +\alpha_{l}\,\beta_{l}\,G_{l\,l}$$

$$+\alpha_{1}\,\sum_{j\neq 1}\beta_{j}\,G_{1\,j}+\alpha_{2}\,\sum_{j\neq 2}\beta_{j}\,G_{2\,j}+\ldots +\alpha_{k}\,\sum_{j\neq l}\beta_{j}\,G_{l\,j},$$

with *G*_*jj*_ the (co)variance elements of the G-matrix. The first line encompasses direct response in the trait while the second line encompasses the correlated selection responses. Factoring out by α_*j*_ gives:

$$\Delta \,\bar{c}=\alpha_{1}\,\left[\beta_{1}\,G_{11}+\sum_{j\neq 1}\beta_{j}\,G_{1\,j}\right]+\alpha_{2}\,\left[\beta_{2}\,G_{22}+\sum_{j\neq 2}\beta_{j}\,G_{2\,j}\right]+\ldots +\alpha_{l}\,\left[\beta_{l}\,G_{l\,l}+\sum_{j\neq l}\beta_{j}\,G_{l\,j}\right],$$

which is the developed form of Johnson et al.’s. (2009) equation. The terms in parentheses are the sum of the direct and correlated genetic changes due to directional selection on the *j^th^* trait of the focal species, i.e., its predicted response to multivariate directional selection. This means that:

$$\Delta \,\bar{c}=\alpha_{1}\,\Delta \,{\bar{z}}_{1}+\alpha_{2}\,\Delta \,{\bar{z}}_{2}+\ldots +\alpha_{l}\,\Delta \,{\bar{z}}_{l}$$

$$\Delta \,\bar{c}=\sum \alpha_{j}\,\Delta \,{\bar{z}}_{j}$$

This exercise clarifies the assumptions behind Johnson et al.’s. (2009) evo-to-eco model. It also illustrates that genetically-based change in a community variable under the influence of a focal species corresponds to the sum of changes of each trait weighted by its respective contribution to the community variable *α_j_*. This is a basically equivalent to a linear selection index (cf. Lin and Allaire, 1977; Nordskog, 1978) except that the values of [*α*_1_, *α*_2_, …*α_l_*] are IIGE parameters estimated with error from data and not fixed *a priori* as in selection indices.
